# CuFe_2_O_4_/MoS_2_ Mixed-Dimensional Heterostructures with Improved Gas Sensing Response

**DOI:** 10.1186/s11671-020-3268-4

**Published:** 2020-02-03

**Authors:** Kenan Zhang, Changchun Ding, Yihong She, Zhen Wu, Changhui Zhao, Baojun Pan, Lijie Zhang, Wei Zhou, Qunchao Fan

**Affiliations:** 1 School of Science, Key Laboratory of High Performance Scientific Computation, Xihua University, Chengdu, 610039 China; 2 School of Microelectronics, Southern University of Science and Technology, Shenzhen, 518055 China; 3 Zhejiang Key Laboratory of Carbon Materials, College of Chemistry and Materials Engineering, Wenzhou University, Wenzhou, 325035 China; 4 National Laboratory for Infrared Physics, Shanghai Institute of Technical Physics, Chinese Academy of Sciences, Shanghai, 200083 China

**Keywords:** MoS_2_, CuFe_2_O_4_ nanotubes, Heterostructures, First-principles calculations, Gas sensors

## Abstract

Mixed-dimensional (2D + *n*D, *n* = 0, 1, and 3) heterostructures opened up a new avenue for fundamental physics studies and applied nanodevice designs. Herein, a novel type-II staggered band alignment CuFe_2_O_4_/MoS_2_ mixed-dimensional heterostructures (MHs) that present a distinct enhanced (20–28%) acetone gas sensing response compared with pure CuFe_2_O_4_ nanotubes are reported. Based on the structural characterizations and DFT calculation results, the tentative mechanism for the improvement of gas sensing performance of the CuFe_2_O_4_/MoS_2_ MHs can be attributed to the synergic effect of type-II band alignment and the MoS_2_ active sites.

## Introduction

Integration of nanostructured materials with dissimilar physical properties is essential for creating multifunctional devices and it has long been a pursuit of nanomaterials science community [1–5]. Two-dimensional (2D) layered materials, such as graphene, g-C_3_N_4_, and MoS_2_, have received broad interdisciplinary attention [6–13], owing to their potential in diverse technologies, including sensors, electronics, optoelectronics, and so on [14–20]. In particular, 2D layered materials provide a new platform for building mixed-dimensional heterostructures (MHs) efficiently with 0D and 1D nanostructures (including quantum dots, nanowires, and nanotubes) [21–29]. According to previous reports, the electrical conductivity, surface activity, and sensing response of MHs can be efficiently tailored by choosing the suitable candidate materials [30–35]. Although most research has been focused on the novel physical properties of MHs based on 2D layered materials, more efforts are still needed to develop the 0D/1D MH-based nanodevices. CuFe_2_O_4_ is an important n-type metal oxide semiconductor with an indirect bandgap in the range of 1.3–1.95 eV [36, 37], which has been considered a promising material for gas sensors because of its naturally abundance, low-cost, environmental friendliness, simple electronic interface, low maintenance, ease of use, and fabrication [38–40]. It is worth noting that the CuFe_2_O_4_-based gas sensors exhibited relatively low responses toward some target gasses (such as ethanol and acetone) [37]. Therefore, it is significant to improve the sensitivity performance of CuFe_2_O_4_-based gas sensors by the reasonable design of MHs. MoS_2_ is one of the most prominent 2D materials possessing a bandgap of 1.2–1.8 eV, because of high surface to volume ratio and highly sensitive to oxygen adsorption allowing their exploration in chemical sensing applications [41].

In this paper, we report a CuFe_2_O_4_/MoS_2_ MHs (1D/2D) for the first time synthesized by two-step method using electrospinning followed by a hydrothermal process. The morphologies, crystal structures, and compositions of the CuFe_2_O_4_/MoS_2_ MHs have been confirmed, and the density function theory (DFT) results further indicate the formation of type-II band alignment in the MHs. The CuFe_2_O_4_/MoS_2_ MHs have obvious advantages for gas sensing, which benefits from the type-II band alignment and active sites in MoS_2_ ultrathin nanosheets. Gas sensing properties of the CuFe_2_O_4_/MoS_2_ MHs are studied in both ethanol and acetone gasses. As was expected, the MHs-based sensor shows substantial improved gas sensing performance compared with pure CuFe_2_O_4_ nanotubes therefore suggesting potential applications of CuFe_2_O_4_/MoS_2_ MHs in highly sensitive gas sensors.

## Method Section

### Synthesis of CuFe_2_O_4_/MoS_2_ MHs

**Fig. 1:**
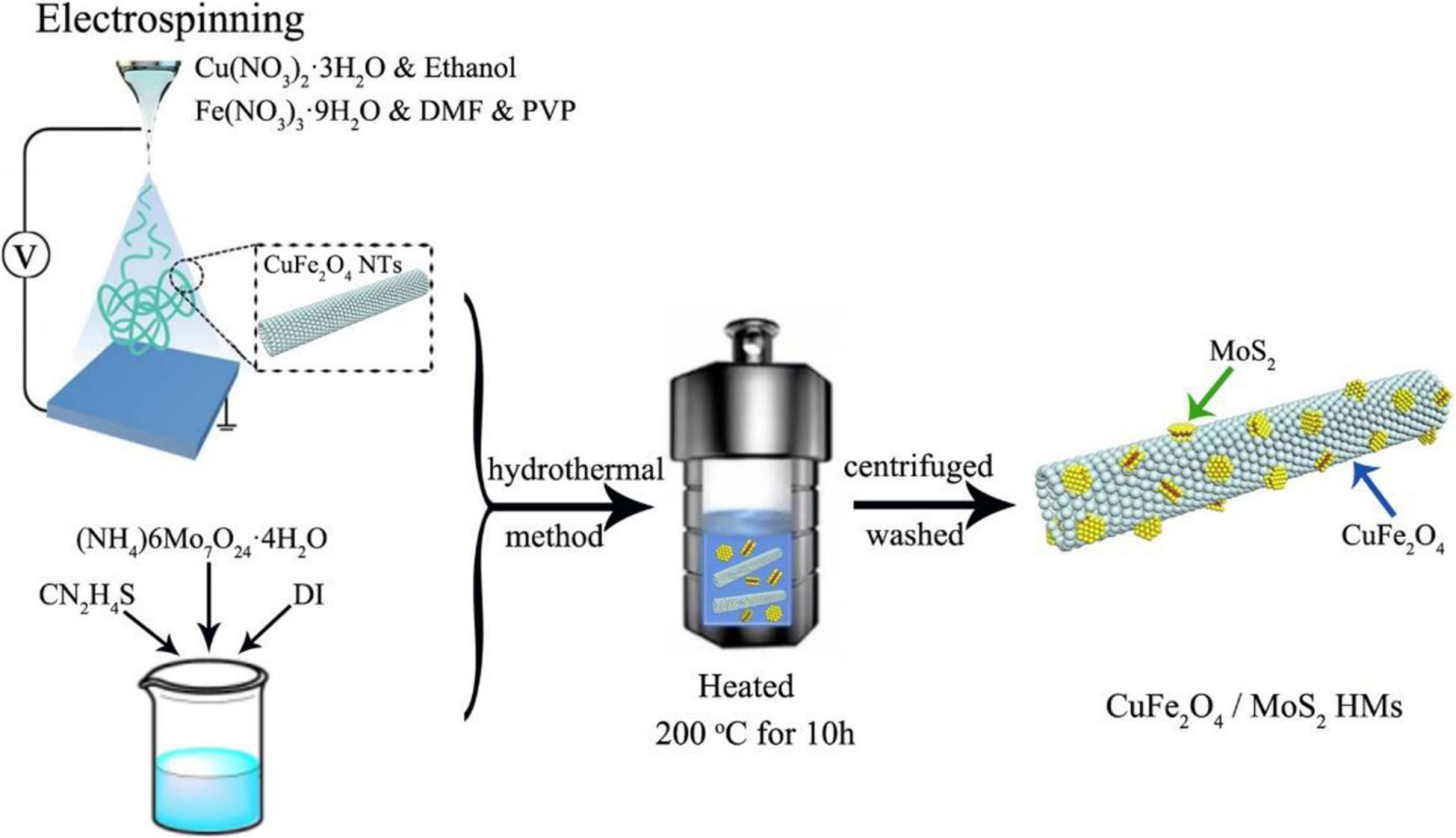
Schematic illustration of the preparation processes of CuFe_2_O_4_/MoS_2_ MHs

The detailed preparation processes of CuFe_2_O_4_/MoS_2_ MHs are shown in Fig. 1. Firstly, the pure CuFe_2_O_4_ nanotubes were pre-synthesized by electrospinning method. Firstly, 0.5 mmol of Cu(NO_3_)_2_·3H_2_O, 1.0 mmol of Fe(NO_3_)_3_·9H_2_O, and 0.68 g of polyvinylpyrrolidone (PVP) were dissolved in 5 mL of ethanol and 5 mL of N,N-Dimethylformamide(DMF). After stirring for 6 h, the above solution was placed in a syringe and injected with a feeding rate of 0.4 mL h^−1^. A DC voltage of 15 kV was applied between the needle tip and stainless-steel mesh with a distance of 18 cm. The as-spun precursor fibers were collected in a tube furnace and maintained at 500 °C for 2 h in air.

The CuFe_2_O_4_/MoS_2_ MHs were synthesized by hydrothermal method in the second step. CuFe_2_O_4_ nanotubes were dispersed in deionized (DI) water (15 mL) via sonication. The (NH_4_)_6_Mo_7_O_24_·4H_2_O and CN_2_H_4_S were then added into the mixture. After stirring for 30 min, the solution was transferred into a 25-mL polytetrafluoroethylene (PTFE) autoclave and kept at 200 °C for 10 h. Finally, the MHs were collected in a centrifuge, washed with DI water and dried at 60 °C.

### Microstructural Characterization

The morphology and structure of pure CuFe_2_O_4_ nanotubes and CuFe_2_O_4_/MoS_2_ MHs were characterized by field emission scanning electron microscopy (FE-SEM, FEI NanoSEM200). X-ray diffraction (XRD) patterns were recorded on a Rigaku Smartlab with Cu Kα radiation operating at 45 kV and 200 mA. Transmission electron microscopy (TEM) measurements were conducted on the JEOL 2100F. The energy dispersive X-ray spectrometer (EDS) was introduced to identify the chemical composition. Raman measurements were performed using a Renishaw inVia at room temperature with a 532-nm excitation laser (2 mW).

### Fabrication and Measurement of Gas Sensors

Gas sensors were fabricated by coating the mixture of the tested materials (pure CuFe_2_O_4_ or CuFe_2_O_4_/MoS_2_ MHs) and DI water onto the interdigitated Au electrode arrays (gap and width are 200 μm) on the SiO_2_/Si substrate. Gas sensing properties of the sensors were measured by using a commercial CGS-4TPs system (Beijing Elite Tech Co., Ltd., China). The response is defined as *R*_a_/*R*_g_, where *R*_a_ is the resistance in atmospheric air and *R*_g_ is the resistance in the tested gas, respectively.

## Results and Discussion

**Fig. 2:**
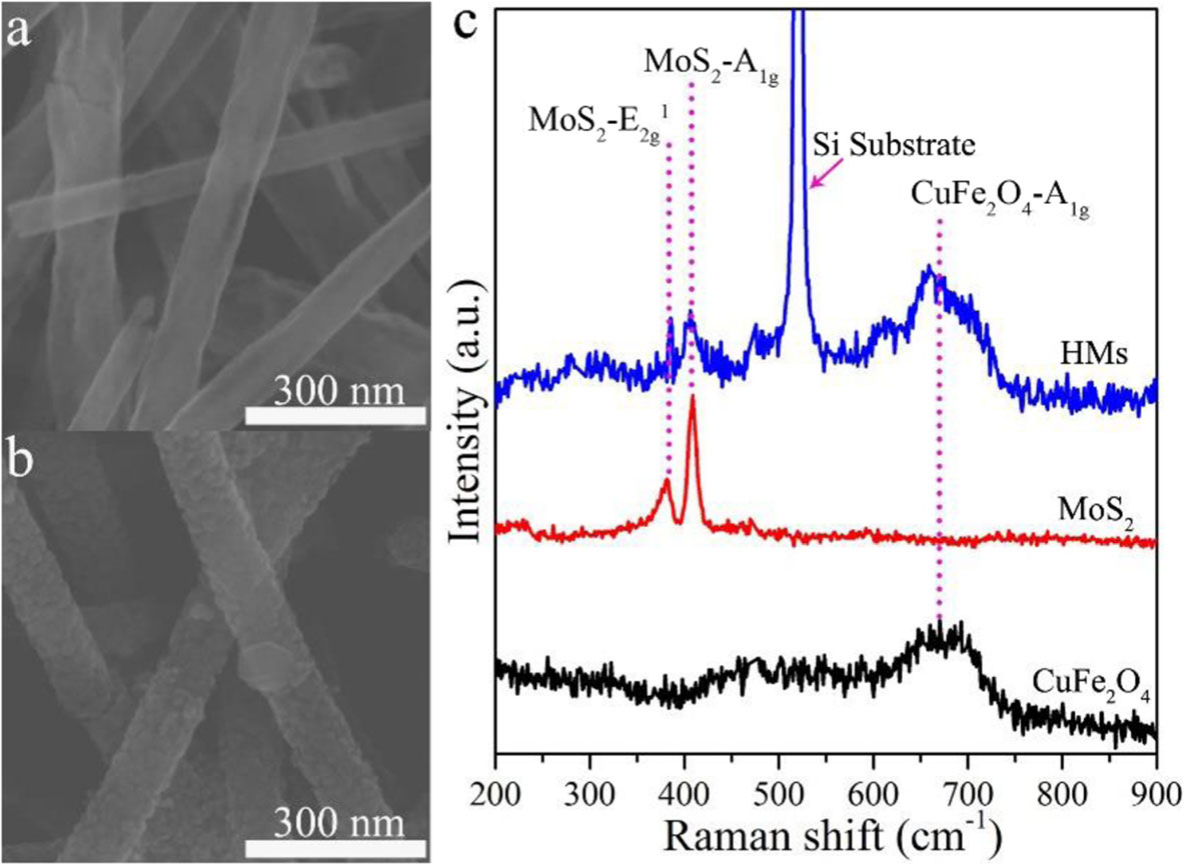
SEM and Raman characterization of CuFe_2_O_4_ and CuFe_2_O_4_/MoS_2_ MHs. FE-SEM images of **a** pure CuFe_2_O_4_ nanotubes and **b** CuFe_2_O_4_/MoS_2_ MHs. **c** Raman spectra of pure CuFe_2_O_4_ nanotubes, pure MoS_2_ nanosheets, and CuFe_2_O_4_/MoS_2_ MHs

The morphologies of pure CuFe_2_O_4_ nanotubes and CuFe_2_O_4_/MoS_2_ MHs are shown in Fig. 2 and Additional file 1: Figure S1. Both of the samples are well-defined tubular nanostructures with several tens of micrometers in length, and 70–150 nm in diameter, which can be confirmed by the cross-section of broken nanotubes (Additional file 1: Figure S1b). The SEM images (Fig. 2a, b) show CuFe_2_O_4_/MoS_2_ MHs still maintains the original tubular structure after the hydrothermal process. And we can see that the CuFe_2_O_4_ nanotubes have a relative smooth surface before compositing with tiny MoS_2_, while the rough surfaces appear in the CuFe_2_O_4_/MoS_2_ MHs. Moreover, Raman spectroscopies were performed to verify the presence of MoS_2_ in the CuFe_2_O_4_/MoS_2_ MHs. The strong vibrational modes of CuFe_2_O_4_ (T_2g_ − 477 cm^−1^, A_1g_ − 685 cm^−1^) and MoS_2_ (${\mathrm{E}}_{2\mathrm{g}}^1$ − 382 cm^−1^, A_1g_ − 409 cm^−1^) can be found in pure CuFe_2_O_4_ nanotube or MoS_2_ nanosheet samples (Fig. 2c). By comparing with the pure CuFe_2_O_4_ nanotubes and MoS_2_ nanosheets (Additional file 1: Figure S2), the Raman vibrational mode of CuFe_2_O_4_ (T_2g_, A_1g_), and MoS_2_ (${\mathrm{E}}_{2\mathrm{g}}^1$, A_1g_) all appeared in the Raman spectrum of CuFe_2_O_4_/MoS_2_ MHs. The position of these four peaks is unchanged, indicating the formation of the composite structure of CuFe_2_O_4_ and MoS_2_ in the CuFe_2_O_4_/MoS_2_ MHs. Meanwhile, the XRD results of pure CuFe_2_O_4_ and CuFe_2_O_4_/MoS_2_ MHs are shows in Additional file 1: Figure S3. It can be seen that the diffraction peaks of CuFe_2_O_4_ are well indexed to the standard JCPDS card (34-0425), revealing that the CuFe_2_O_4_ belongs to a body-centered tetragonal structure. The XRD pattern of the CuFe_2_O_4_/MoS_2_ is superimposed by the diffraction peaks of CuFe_2_O_4_ and MoS_2_, respectively (the standard JCPDS card of CuFe_2_O_4_ (34-0425) and MoS_2_ (06-0097)), and there is no characteristic peak for impurity in the XRD pattern, indicating that the composite is consisted by the CuFe_2_O_4_ and MoS_2_ only.

**Fig. 3:**
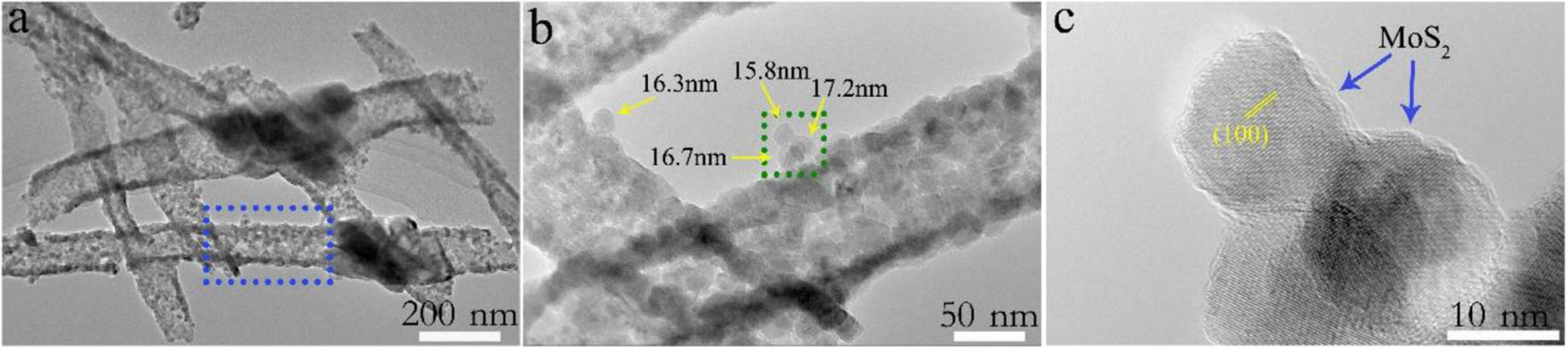
TEM characterization of CuFe_2_O_4_/MoS_2_ MHs. Low-resolution TEM image of **a** CuFe_2_O_4_/MoS_2_ MHs and **b** partial zooming panel **a** in the dotted line. **c** HRTEM image of the region in the dotted line in the **b**

**Fig. 4:**
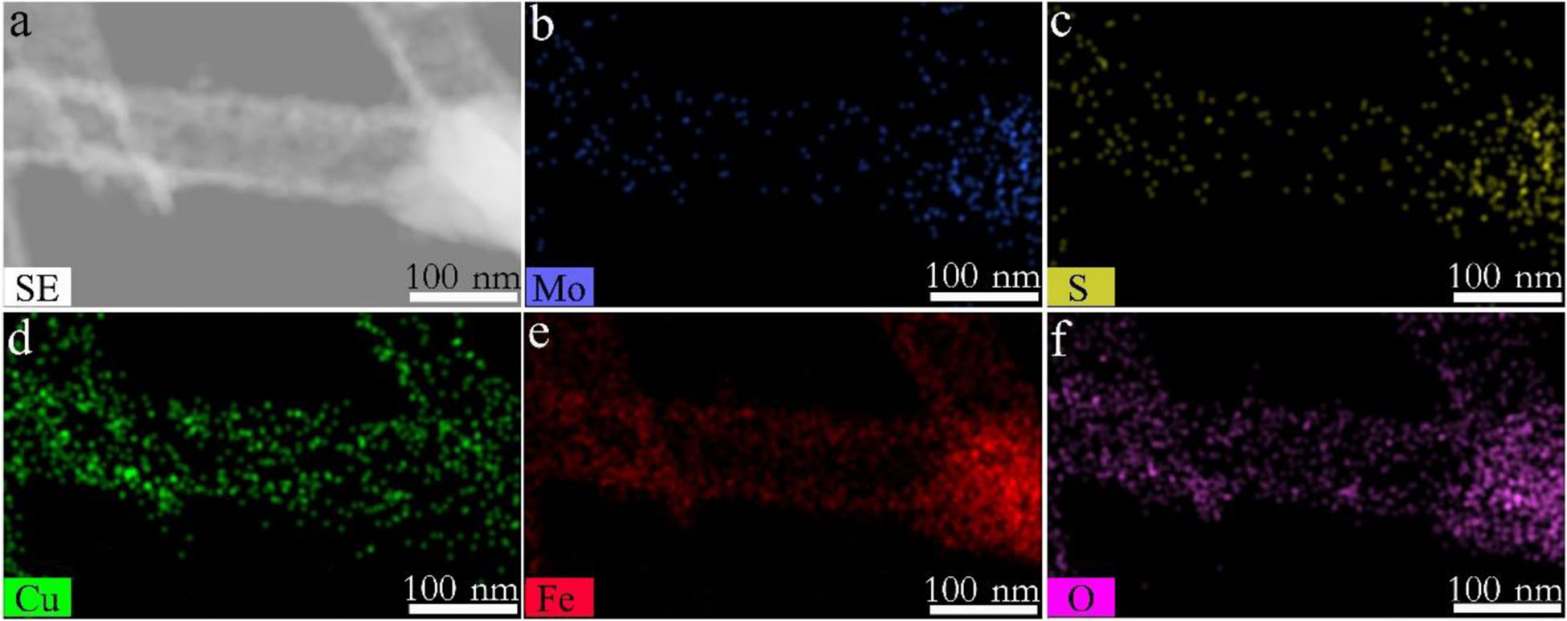
EDS result of CuFe_2_O_4_/MoS_2_ MHs. **a** SEM image of sample in dotted line of Fig. 3a. **b**–**f** The in-suit EDS intensity map of Mo, S, Cu, Fe, and O, respectively

To further characterize the microstructure of CuFe_2_O_4_/MoS_2_ MHs, TEM observations were carried out, as shown in Fig. 3 a. The low-resolution TEM images (Fig. 3b) show that the surfaces of CuFe_2_O_4_ nanotubes are uniformly covered with many hexagonal nanosheets 15–20 nm in diameter. Figure 3 c gives the high-resolution TEM (HRTEM) images of tiny nanosheets marked in Fig. 3b. The lattice fringes spacing of 0.27 nm can be corresponded to the (100) plane of MoS_2_. In addition, the morphology and size of MoS_2_ can be tailored by adjusting the hydrothermal reaction conditions (Additional file 1: Figure S2). Selected area electron diffraction (SAED) pattern also reveals the hexagonal symmetry for the layered MoS_2_ (Additional file 1: Figure S4). To demonstrate the distribution of MoS_2_ nanosheets on the surface of CuFe_2_O_4_ nanotubes, the in situ EDS elemental mapping images of CuFe_2_O_4_/MoS_2_ MHs (marked in Fig. 3b) are performed as shown in Fig. 4. The homogeneous distribution of Mo, S, Cu, Fe, and O elements indicates that a large number of MoS_2_ nanosheets are uniformly dispersed in CuFe_2_O_4_/MoS_2_ MHs.

**Fig. 5:**
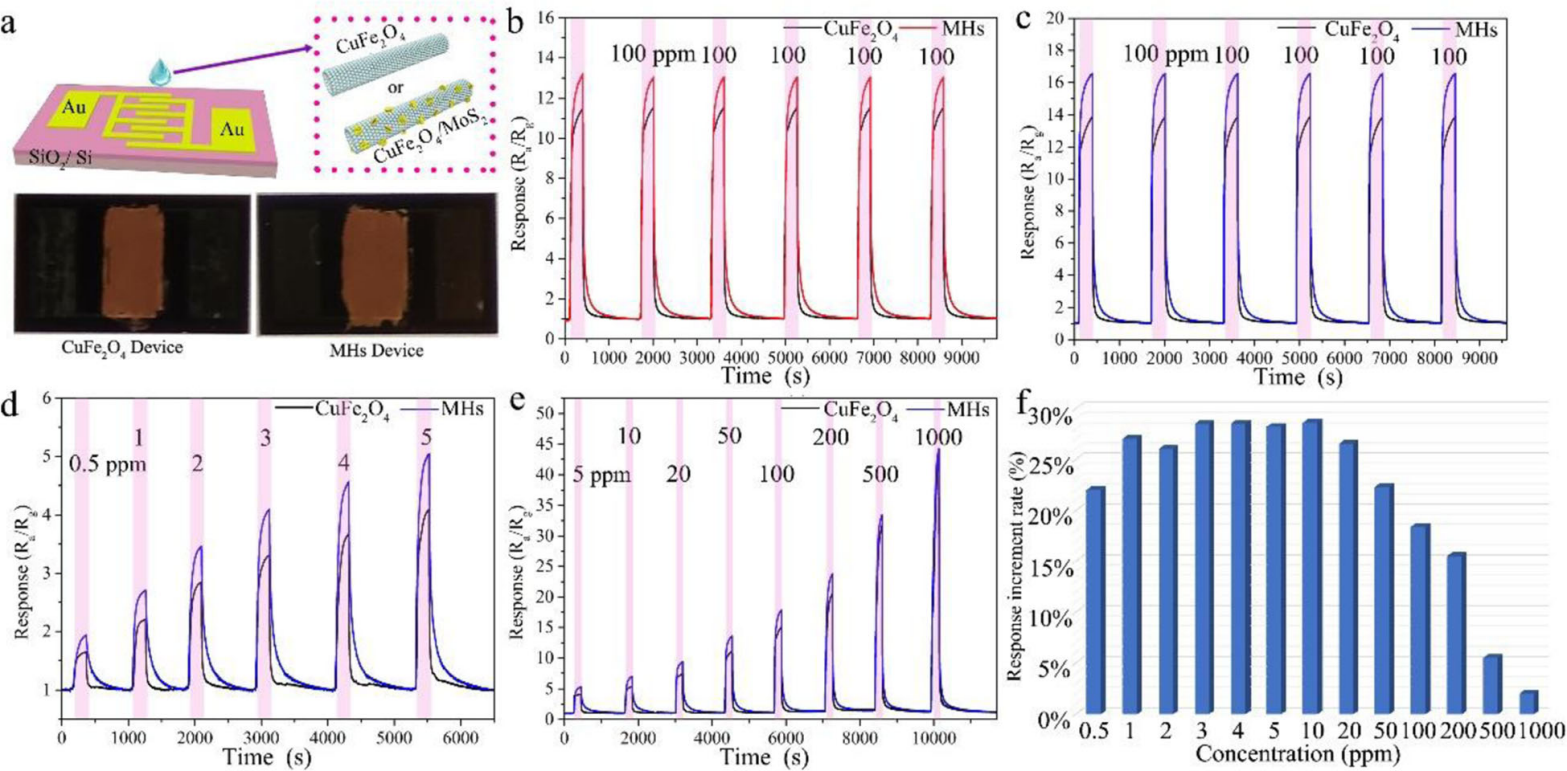
Sensing measurements of CuFe_2_O_4_/MoS_2_ MHs. **a** Fabricated diagram of gas sensor and photos of fabricated gas sensor (CuFe_2_O_4_ nanotubes and CuFe_2_O_4_/MoS_2_ MHs). Sensing reproducibility of the CuFe_2_O_4_ nanotubes and CuFe_2_O_4_/MoS_2_ MHs gas sensor to 100 ppm **b** ethanol and **c** acetone. **d**, **e** Dynamic response-recovery curves of CuFe_2_O_4_ nanotubes and CuFe_2_O_4_/MoS_2_ MHs gas sensors at different acetone concentrations. **f** The response increment rate of CuFe_2_O_4_/MoS_2_ MHs device relative to pure CuFe_2_O_4_ nanotube device at different acetone concentrations

In order to investigate their gas sensing properties, the pure CuFe_2_O_4_ nanotubes and CuFe_2_O_4_/MoS_2_ MHs gas sensors were fabricated as shown in Fig. 5 a and Additional file 1: Figure S5. Figure 5b and c preset the response-recovery curves of pure CuFe_2_O_4_ nanotubes and CuFe_2_O_4_/MoS_2_ MHs gas sensors toward 100 ppm ethanol and acetone (6 cycles), respectively. After compositing with the MoS_2_ nanosheets, it can be seen that the CuFe_2_O_4_/MoS_2_ MHs sensor shows positive responses on exposure to both ethanol and acetone, which are about 18–20% higher than those of pure CuFe_2_O_4_ nanotubes. Evidently, the CuFe_2_O_4_/MoS_2_ MHs sensor exhibits consistent sensing responses even after 6 cycles, indicating the good reversibility and repeatability. Figure 5d and e give the dynamic transient response curves of pure CuFe_2_O_4_ nanotubes and CuFe_2_O_4_/MoS_2_ MHs gas sensors to various acetone concentrations (0.5–1000 ppm). The CuFe_2_O_4_/MoS_2_ MHs sensor exhibits improved response to each acetone concentration (Fig. 5f). In particular, the percentage of improvement in acetone response exceeds 20% at acetone concentrations not higher than 50 ppm. It is noticeable that the acetone responses improved about 18% even at 0.5 ppm. That means the CuFe_2_O_4_/MoS_2_ MHs are more sensitive to acetone in contrast with pure CuFe_2_O_4_.

**Fig. 6:**
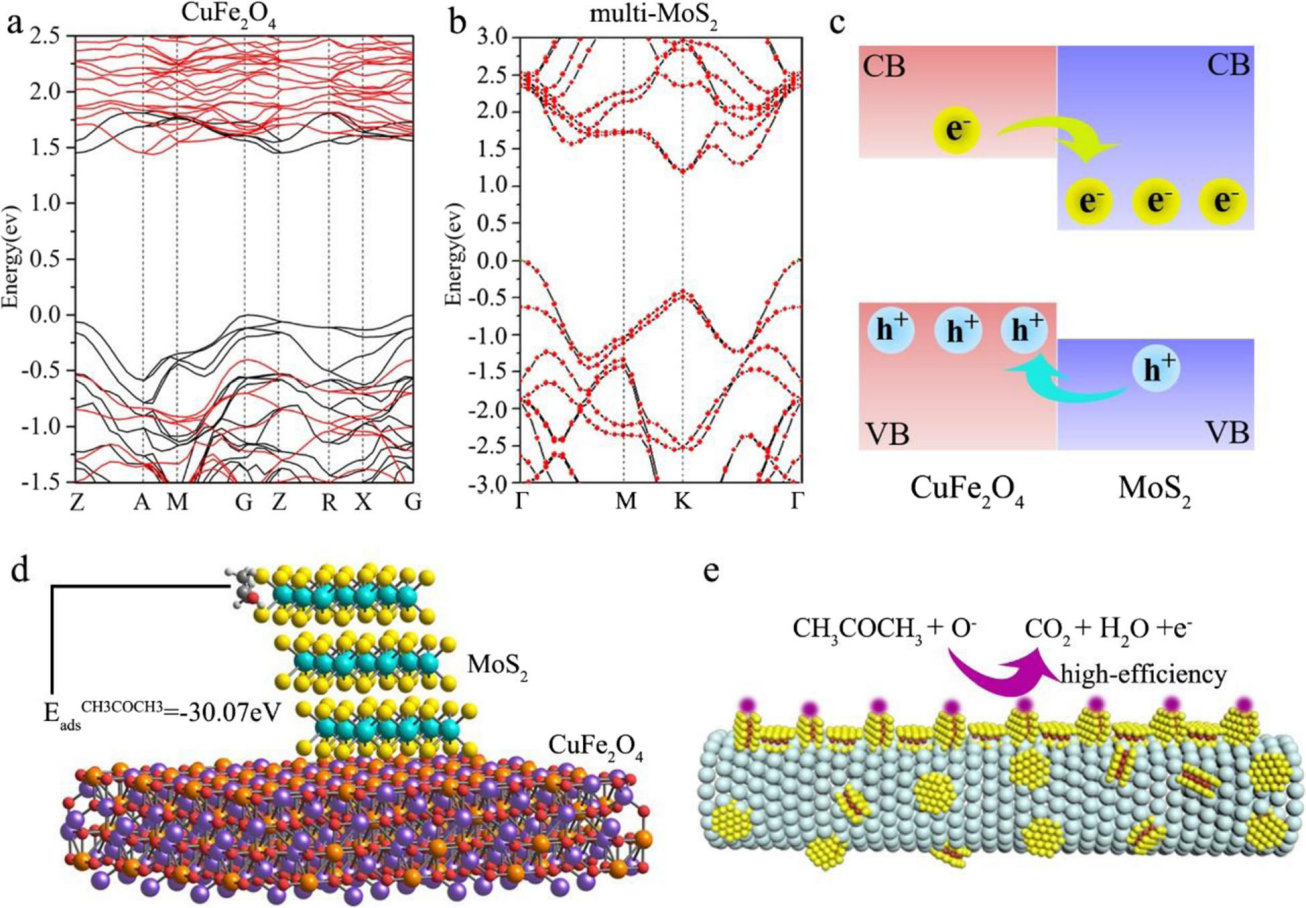
DFT results of CuFe_2_O_4_/MoS_2_ MHs. Electronic structures of **a** CuFe_2_O_4_ nanotubes and **b** multilayer MoS_2_. **c** Schematic illustrations of the type-II band alignment in CuFe_2_O_4_/MoS_2_ MHs. **d** The edge adsorption energy for CH_3_COCH_3_ molecules on CuFe_2_O_4_/MoS_2_ MHs. **e** Model for the CuFe_2_O_4_/MoS_2_ MHs in acetone vapor

To probe the important role of MoS_2_ nanosheets in the gas sensing reaction, the electronic band structures of CuFe_2_O_4_ and multilayer MoS_2_ were calculated respectively by using DFT (Fig. 6a, b). The indirect bandgap of CuFe_2_O_4_ and multilayer MoS_2_ is about 1.3 eV and 1.2 eV, respectively. According to the results, the band alignment of CuFe_2_O_4_/MoS_2_ MHs is drawn in Fig. 6c, which forms a type-II band alignment. The improvement of sensor response manifested in changes in the electrical resistance (*R*_a_/*R*_g_) in the presence of air or target gas. Because of the type-II band alignment, the electron-hole pairs can be separated effectively at the heterojunction interface. Holes remain within the CuFe_2_O_4_ nanotubes, while most electrons will be injected into MoS_2_ layers. When the pure CuFe_2_O_4_ or CuFe_2_O_4_/MoS_2_ MHs sensors are exposed to air, oxygen molecules will adsorb on the surface of sensors to generate oxygen species (O_2_^−^, O^−^, and O^2−^). Meanwhile, the free electrons transfer from CuFe_2_O_4_ or CuFe_2_O_4_/MoS_2_ MHs to oxygen species at sensors surface lead to the decreases of electrical resistance (*R*_*a*_). In the case of target gas detection, the reaction of adsorbed oxygen species and target molecules will occur on the sensor surface (e.g., CH_3_COCH_3_ + 8O^−^ → 3CO_2_ + 3H_2_O + 8e^−^) and release free electrons to the CuFe_2_O_4_ or CuFe_2_O_4_/MoS_2_ MHs. Thus, the sensor resistance (*R*_g_) decreases in target gas. It is noteworthy that the MoS_2_ edges offer high density of potential active sites for reduction reaction [42–44]. Figure 6 d shows the calculated adsorption energy of CH_3_COCH_3_ on CuFe_2_O_4_/MoS_2_ MHs by using the DFT method. The adsorption energy for CH_3_COCH_3_ molecules over the edge of CuFe_2_O_4_/MoS_2_ MHs is − 30.07 eV (very small). That means the edge of CuFe_2_O_4_/MoS_2_ MHs are active sites for CH_3_COCH_3_ molecules. Benefiting from the active sites in MoS_2_ nanosheets, the CuFe_2_O_4_/MoS_2_ MHs obtained free electrons more efficiently compared with pure CuFe_2_O_4_ (Fig. 6e). The positive effect is more obvious in low target gas concentration. While the improved gas response performance is limited in the extra-high concentrations due to the limited active sites.

## Conclusions

We report a novel CuFe_2_O_4_/MoS_2_ MHs and the obvious improvement of sensing performance for acetone. The CuFe_2_O_4_/MoS_2_ MHs are confirmed by Raman, SEM, XRD, TEM, and EDS results. The coupling interactions between CuFe_2_O_4_ and MoS_2_ lead to the formation of type-II heterostructures, which is verified by DFT results. The practical gas sensor devices were fabricated based on CuFe_2_O_4_/MoS_2_ MHs and shows the high sensitivity and excellent repeatability. A sensing enhancement is also seen with ethanol gas. The enhancement of gas sensing properties of the CuFe_2_O_4_/MoS_2_ MHs can be attributed to the effect of type-II band alignment and the MoS_2_ active sites. We believe that our studies will be valuable for the various applications of mixed-dimensional heterostructures.

## Supplementary information

**Additional file 1: Figures S1–S5.** Additional experimental details, SEM, TEM, SAED and XRD results.

## Data Availability

All data are fully available without restriction.

## Abbreviations

2D: Two-dimensional
DFT: Density function theory
EDS: Energy dispersive X-ray spectrometer
MHs: Mixed-dimensional heterostructures
SEM: Scanning electron microscope
TEM: Transmission electron microscopy
