# Supplementary material for: CuFe2O4/MoS2 Mixed-Dimensional Heterostructures with Improved Gas Sensing Response
Source: Nanoscale Res Lett. 2020 Feb 3;15:32. doi: 10.1186/s11671-020-3268-4 (PMC6997305; doi:10.1186/s11671-020-3268-4)
Supplement: Supplementary file 1 — Additional file 1: Figures S1–S5. Additional experimental details, SEM, TEM, SAED and XRD results. [file 11671_2020_3268_MOESM1_ESM.docx]

**Additional file**

CuFe_2_O_4_ / MoS_2_ Mixed-Dimensional Heterostructures with Improved Gas Sensing Response

Kenan Zhang^1^*, Changchun Ding^1^, Yihong She^1^, Zhen Wu^1^, Changhui Zhao^2^*, Baojun Pan^3^, Lijie Zhang^3^, Wei Zhou^4^, Qunchao Fan^1^*

E-mail:

Kenan Zhang: [knzhang@mail.sitp.ac.cn](mailto:knzhang@mail.sitp.ac.cn);

Changchun Ding: ccding626@163.com;

Yihong She: 499810935@qq.com;

Zhen Wu: wuzhen1333@163.com;

Changhui Zhao: zhaoch@sustech.edu.cn;

Baojun Pan: 1652315036@qq.com;

Lijie Zhang: zlj4165@126.com;

Wei Zhou: zhouwei@mail.sitp.ac.cn;

Qunchao Fan: fanqunchao@mail.xhu.edu.cn.

1 School of Science, Key Laboratory of High Performance Scientific Computation, Xihua University, Chengdu 610039, China.

2 School of Microelectronics, Southern University of Science and Technology, Shenzhen 518055, China.

3 College of Chemistry and Materials Engineering, Wenzhou University, Zhejiang Key Laboratory of Carbon Materials, Wenzhou 325035, China.

4 National Laboratory for Infrared Physics, Shanghai Institute of Technical Physics, Chinese Academy of Sciences, Shanghai 200083, China.


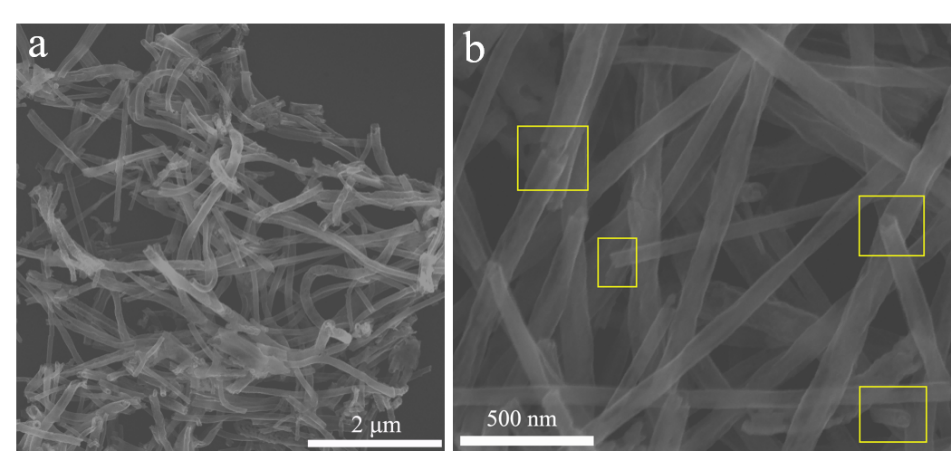


Figure S1. FE-SEM images of pure CuFe_2_O_4_ nanotubes. The cross-section of broken nanotubes is shown in the yellow line of panel S1(b).


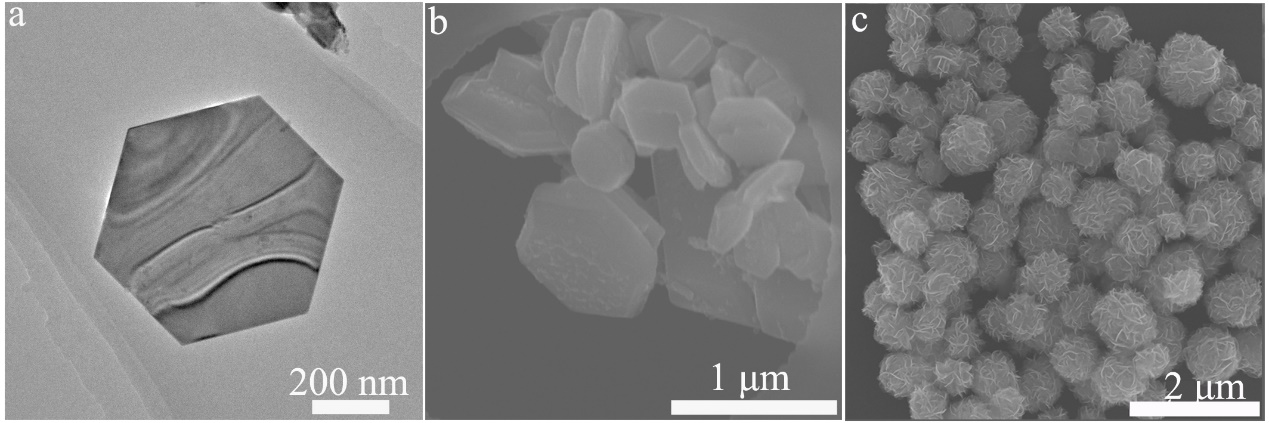


Figure S2. The morphology of MoS_2_ synthesized by hydrothermal method. The small MoS_2_ nanosheets were synthesized in low temperature and precursor concentration (a). With the increasing of the temperature and precursor concentration, the MoS_2_ nanosheets diameter and thickness increased (b, 220 °C). The morphology of MoS_2_ is self-assembled by the MoS_2_ nanosheet to form a fluffy ball-like structure with high temperature and precursor concentration (c, 250 °C and double the concentration of precursor).


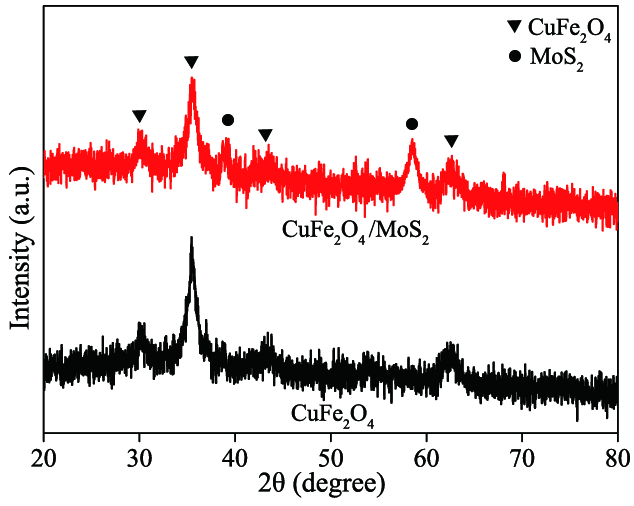


Figure S3. XRD patterns of CuFe_2_O_4_ and CuFe_2_O_4_/MoS_2_.


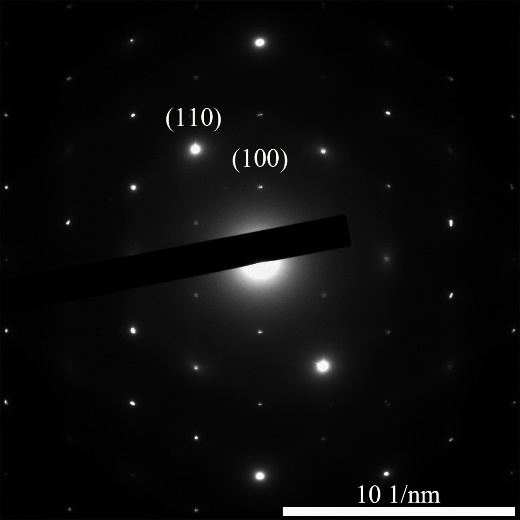


Figure S4. Typical SAED pattern of a MoS_2_ nanosheets in Figure S2a


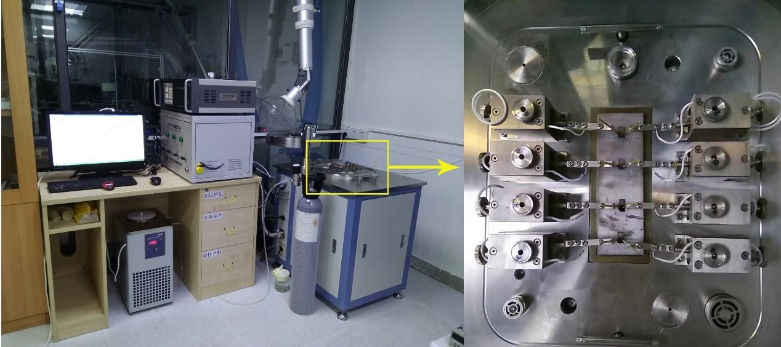


Figure S5. The photograph of sensing test system and test bench.
